# Randomized pilot study of an individualized multimodal exercise, nutrition, and behavior intervention in breast cancer patients treated with ovarian function suppression: protocol proposal for The OvS Breast ENBI Project

**DOI:** 10.3389/fonc.2025.1622622

**Published:** 2025-10-27

**Authors:** Blanca Herrero López, Mónica Castellanos Montealegre, Candelaria Soulas, Begoña Arbulo Rufrancos, María Luisa García-Ontiveros Cuéllar, María del Monte Millán, Lucía Villarejo López, Sara López-Tarruella Cobo, Yolanda Jerez Gilarranz, Isabel Echavarría Díaz-Guardamino, Pablo Jara, Miguel Martín Jiménez, Tatiana Massarrah Sánchez, María Jesús Martínez Beltrán, Julio César de la Torre Montero, Soraya Casla Barrio

**Affiliations:** ^1^ Department of Medical Oncology, Breast Cancer Unit, Hospital General Universitario Gregorio Marañón, IiSGM, Madrid, Spain; ^2^ Centro Ejercicio y Cáncer, Madrid, Spain; ^3^ Department of Psychiatry and Clinical Psychology, Hospital General Universitario Gregorio Marañón, Madrid, Spain; ^4^ Department of Medical Oncology, Translational Research Unit, Hospital General Universitario Gregorio Marañón, IiSGM, Madrid, Spain; ^5^ Universidad Pontificia Comillas, San Juan de Dios Nursing and Physiotherapy School, Ciempozuelos, Madrid, Spain; ^6^ San Juan de Dios Foundation, Madrid, Spain

**Keywords:** breast cancer, physical exercise, nutrition, behavioral, psycho-oncology, clinical trial

## Abstract

**Introduction:**

Breast cancer (BC) is the most common malignancy among women in Spain and worldwide. Ovarian function suppression (OFS) is recommended as an adjuvant strategy for high-risk hormone receptor (HR)-positive premenopausal BC patients. However, OFS is associated with unfavorable changes in body composition, weight gain, and adverse cardiorespiratory and emotional effects. Multimodal, individualized interventions integrating nutrition, exercise, and psycho-oncological support have demonstrated safety and efficacy in promoting healthy body composition, weight control, and cardiorespiratory fitness (CRF). This study aims to evaluate the impact of a personalized multicomponent intervention in premenopausal BC patients receiving adjuvant OFS.

**Methods and analysis:**

We present the ENBI project study protocol proposal as a single-center, open-label, 2:1 randomized pilot study designed to assess the effects of a 12-week individualized program (nutrition, exercise, and psycho-oncological support) versus the World Health Organization (WHO) healthy lifestyle recommendations. Participants are premenopausal HR-positive BC patients undergoing adjuvant OFS. The study duration is estimated at 15 months. The primary endpoint is weight and body composition change, measured via scale and bioelectrical impedance analysis at baseline, post-intervention, and at 6- and 12-month follow-up. Secondary outcomes include CRF, cardiac variability, muscle strength, physical function, laboratory parameters, patient-reported outcomes (quality of life, fatigue, physical activity), systemic therapy-associated adverse events, and nutritional and psychological status. Exploratory outcomes include inflammatory markers (C-reactive protein, tumor necrosis factor alpha, adiponectin) and oncostatin-M. Due to the lack of prior data, the sample size was pragmatically set based on the Hospital General Universitario Gregorio Marañón (HGUGM) Tumor Board registry, with an estimated recruitment of 30 patients.

**Ethics and dissemination:**

The HGUGM Drug Research Ethics Committee (CEIm) has approved the study protocol. Results will be presented at national and international conferences and published in a peer-reviewed journal.

**Trial registration number:**

NCT06727487

## Introduction

Breast cancer (BC) is the most common and incident cancer among Spanish women. Hormone receptor (HR) - positive BC is the most prevalent subtype, accounting for approximately 75% of all BC cases (1).

Ovarian function suppression (OFS) combined with endocrine therapy (gonadotropin-releasing hormone agonists (GnRHa) administered along with tamoxifen (TAM) or aromatase inhibitors (AI)) have demonstrated a benefit in terms of relapse-free survival (RFS) and overall survival (OS) in premenopausal women diagnosed with HR-positive BC, particularly among younger patients with high-risk pathological features (2–4). Accordingly, international clinical guidelines recommend OFS in such clinical scenarios (5, 6).

However, relevant cardiorespiratory, metabolic, sexual, as well as emotional adverse events have been associated with OFS (2, 7): Early body composition changes such as fat mass and weight increases (8, 9) have been directly associated to higher BC relapse risk (10, 11) along with a reduced adherence to endocrine treatment (12).

Body fat gain, along with muscle mass loss, results in a proinflammatory (13) and a dysfunctional immune system, which has been linked to cardiovascular disease development and poor outcomes in cancer patients (14–16). In addition, chronic inflammation has been linked to cancer development (17). Within this framework, it should be noted that skeletal muscle acts as an endocrine organ whose contraction releases myokines (cytokines or peptides) that can exert an anti-inflammatory action (18).

Current evidence suggests that multimodal, individualized interventions combining nutrition, exercise, and psycho-oncological support tailored to physiological needs, baseline physical condition and potential adverse events are safe (19). This integrative approach has been shown to be effective in achieving a healthy weight and body composition in BC patients (20, 21), may contribute to improve immune function (22, 23) and enhance cardiorespiratory fitness (CRF) leading to a positive impact on quality of life (QoL) (20, 23).

On the other hand, securing high patient adherence to these programs is critical, and, in this context, psycho-oncological support and behavioral interventions would be able to stimulate lifestyle changes in BC patients and improve adherence rates (24, 25).

To date, there is no consensus on optimal exercise prescription or nutritional strategies in premenopausal BC patients undergoing OFS. Therefore, we consider that tailored interventions to patients´ specific characteristics and factors influencing adherence remain essential. This pilot study evaluates the feasibility and preliminary effects of a personalized multimodal program (nutrition, exercise, and psycho-oncology) for premenopausal BC patients receiving adjuvant OFS.

## Methods and analysis

### Main outcomes

The primary outcome of the study is to evaluate the impact of the combined intervention on weight and body composition. Secondary outcomes aim to evaluate the impact of the combined intervention program on exercise capacity, nutritional status, psychological outcomes, and treatment-related side effects.

### Study design

**Figure 1:**
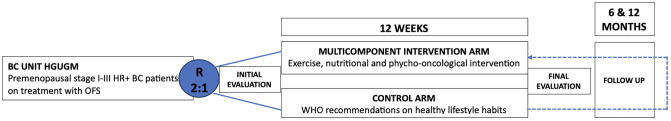
ENBI study design scheme. Patients are randomly assigned to a 12 week-intervention arm (2-day per week exercise training session, 3 one-to-one nutritional intervention sessions and 3 group psycho-oncological support sessions) or control arm.

This is a proposed study protocol of a single-center, open-label, 2:1 randomized pilot trial (ClinicalTrials.gov: NCT06727487). Premenopausal HR-positive BC patients undergoing adjuvant OFS treatment will be randomized to receive either a 12-week individualized exercise, nutritional and psycho-oncological intervention (intervention group) or WHO healthy lifestyle recommendations (control group). Cross-over is allowed for patients in the control group after the final follow-up (**Figure 1**). See Standard Protocol Items Recommendations for Interventional Trials (SPIRIT) checklist at **Supplementary Appendix 1**.

### Randomization and masking

An independent researcher will generate the allocation sequence using a computer-based randomization program and a concealed allocation list. No stratification factors are applied due to the pilot nature of the study. Study oncologists at HGUGM will enroll participants and ensure that all inclusion criteria are fulfilled and no exclusion criteria are present. The group assignment will not be revealed to participants until enrollment is confirmed.

This is an open-label trial; no blinding is applied to participants, providers, or outcome assessors.

### Study setting and participants

The trial is conducted at Hospital General Universitario Gregorio Marañón (HGUGM), in collaboration with *Ejercicio y Cáncer Center* and *Universidad Pontificia Comillas*.

Eligible patients should meet the following inclusion and exclusion criteria:

Inclusion criteria

- At least 18 and up to 45 years of age at the time of consent.
- Histologically confirmed HR-positive stage I to III invasive BC.
- Premenopausal status is clinically defined as a patient who maintains menstruation prior to chemotherapy initiation if he has taken place and/or regular menstruation at the time of consent.
- Completed locoregional treatment (surgery and radiotherapy).
- Ongoing adjuvant treatment, including OFS drugs, is expected to be maintained for at least 4 months at the time of consent.
- Functional status by the Eastern Cooperative Oncology Group (ECOG) scale 0-1.
- Ability to understand and give informed consent (IC).
- Exclusion criteria
- Any medical contraindication to exercise practice.
- Any American Thoracic Society (ATS) contraindications for cardiopulmonary exercise testing (26).
- Active metastatic BC or other concurrent cancer diagnosis at the time of consent.
- Pregnant or breastfeeding women.
- Alcohol or other drug abuse (excluding smoking) is defined as a pattern of usual consumption that results in physical, mental, or social functioning impairment.
- Any condition that makes the patient ineligible is based on the investigator’s criteria.

### Intervention

This study is expected to run for 1 year and 3 months: Study intervention has a duration of 12 weeks, and the follow-up period will last 12 months from the end of the intervention period.

Patients allocated to the control group will receive standard WHO lifestyle guidance and virtual materials for BC survivors. Patients assigned to the multidisciplinary intervention program will also be provided with the same virtual material and will follow a 12-week combined intervention program (see TIDieR guide recommendations for each intervention at **Supplementary Appendix 2**):

- A combined 2-day per week exercise training session guided by an exercise physiologist specialized in oncology: It will include cardiovascular exercise planned at 55-100% of Heart Rate Reserve (HRR) which will be controlled by pulsometer and physical condition-adapted strength exercise planned at 40-70% of One-repetition maximum (1 RM) estimated from the 5-RM test using the Reynolds equation.). Exercise intensity will also be assessed through the Borg perception of effort scale (27). Each session incorporated neuromotors, strength, and cardiovascular exercises, beginning with low-intensity activities and progressively increasing in intensity and difficulty every three weeks (by increments of 5%). Intensity was increased only if participants were capable of achieving the prescribed level; otherwise, the workload was maintained and adapted in the following session. All variables related to adherence and safety—including attendance, adaptations in intensity or activities, and dropouts—were systematically recorded.
- Three one-to-one nutritional intervention sessions guided by a cancer expert nutritionist: Nutritional behavior will be evaluated to stablish effective methods to achieve healthier diet habits.
- Three group psycho-oncological support sessions: Emotional status and motivation will be assessed; the main objective of this intervention consists of stimulating motivation to change lifestyle habits toward healthier ones and increasing adherence to them (28).

To promote participants’ retention, regular contact and reminders will be used. If a participant discontinues the intervention, efforts will be made to collect primary outcome data at scheduled timepoints.

### Efficacy assessment

The primary endpoint is to evaluate the impact of the combined intervention on weight and body composition. This endpoint will be assessed by the following variables: weight, height, body mass index (IMC), waist and hip circumferences, waist-to-hip ratio (WHR), fat mass, lean mass, and extracellular water, which will be obtained with Tanita BC-601 Gold weight scale and bioelectrical impedance analysis, as well as a measuring tape.

The secondary endpoints include evaluating the impact of combined intervention on 1) CRF estimated by Bruce test and maximum oxygen uptake (VO2max), 2) cardiac variability assessed by resting and maximal heart rate (HR) (29), 3) endurance performance measured by capillary lactate levels at rest, maximum effort and two minutes after maximum effort (29), 4) muscle strength evaluated by upper body dynamometry and five-maximal repetition text in shoulder press and squat, 5) physical function assessed by 30-second sit-to-stand test and 6-minute walking test, and 6) potentially modifiable laboratory parameters such as complete blood count, glucose levels, complete liver and renal function panels, electrolyte concentrations, protein and albumin levels, complete lipid profile and thyroid function.

Patient reported outcomes (PROs) are also considered secondary endpoints. They will be assessed and collected using validated questionnaires: 1) EuroQoL-5D (30) (QoL), 2) FACIT-Fatigue (31) (fatigue), 3) International Physical Activity Questionnaire (32) (IPAQ) (physical activity level), 4) Rosenberg self-esteem scale (33) (self-esteem), 5) Hospital Anxiety and Depression Scale (34) (HADS) (anxiety and depression levels).

Concerning systemic therapy-associated adverse events, investigators will evaluate and grade them according to CTCAE version 5 as secondary endpoints (lymphedema, peripheral neuropathy, arthralgias, irritability, insomnia, vaginal dryness, decreased libido, hot flashes).

Finally, nutritional and psychological status are also included as secondary endpoints. Nutritional status will be assessed by a 3-day nutritional record (27, 35), PREDIMED Mediterranean Diet Adherence Score (36) and compliance with WCRF/AICR cancer prevention recommendations (37, 38). Positive and negative affect scale (PANAS) (39), transtheoretical model of physical activity change questionnaire (40, 41) and MOS social support survey (42, 43) will serve to evaluate psychological status.

**Figure 2:**
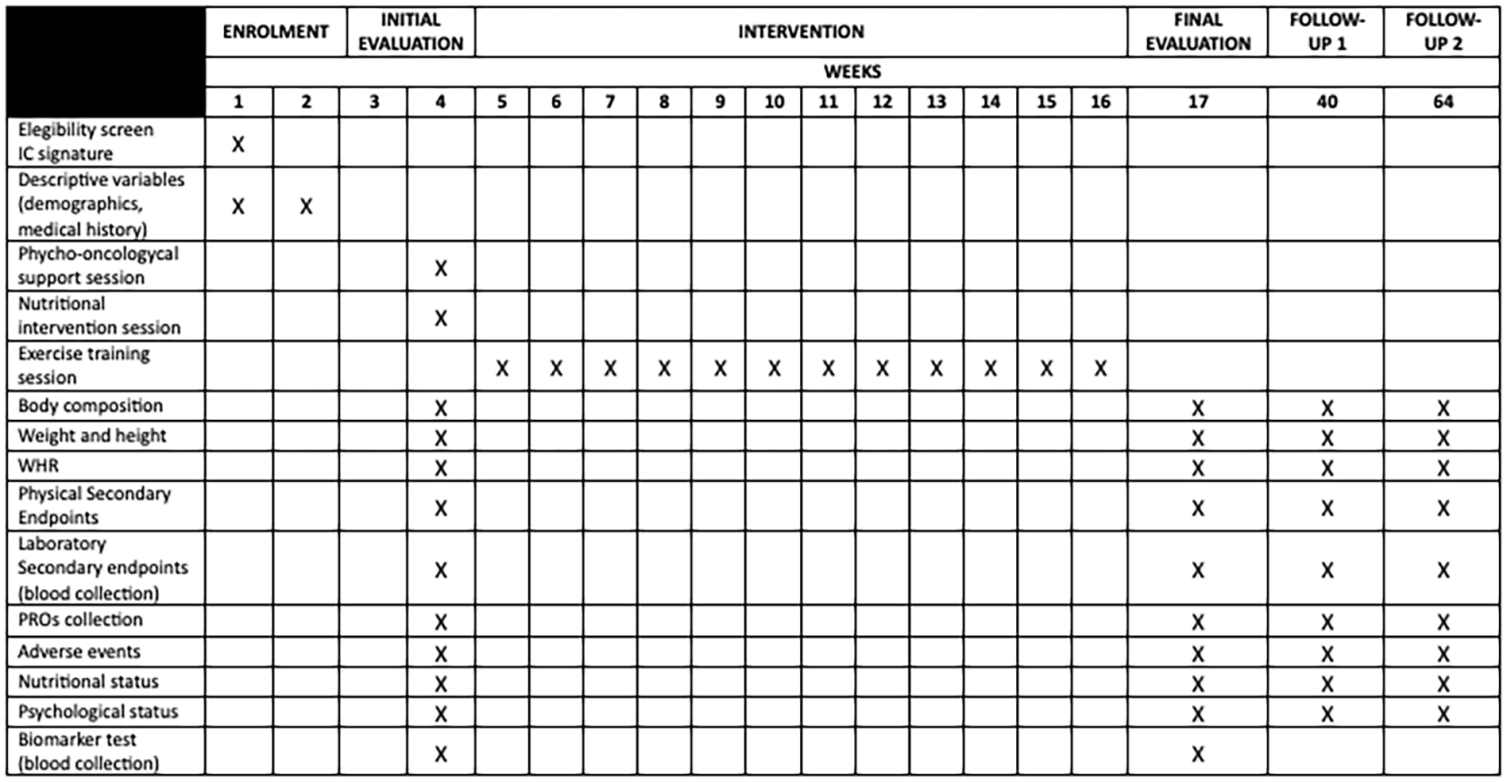
Schedule for patient enrolment, combined intervention, and assessment of the ENBI study.

Primary and secondary endpoints will be assessed immediately before the 12-week intervention period and at the end of it. They will be also evaluated at 6- and 12-months follow up timepoints (see **Figure 2** study schedule).

### Biomarker study

The biomarker test proposed for this study aims to evaluate the potential impact of the combined intervention on pro-inflammatory markers (C-reactive protein (CRP), tumor necrosis factor alpha (TNF-α)), anti-inflammatory markers (adiponectin (13)) and oncostatin-M (multifunctional myokine that has been related to apoptosis stimulation in BC cell lines (44)).

### Study schedule

Assessments and enrollment schedule are summarized in **Figure 2**. We define week 1 to 3 as the moment in which patients are enrolled and demographic information is collected. Initial and final assessments take place the week before and after the 12-week intervention period (week 4 and 17), respectively. Blood for secondary endpoints and biomarker analysis is collected on week 4 and 17. On week 40 and 64, 6 and 12 months after the 12-week intervention respectively, a new general assessment (physical, nutritional and psycho-oncological evaluation) along with blood collection for secondary endpoints take place.

### Statistical analysis

Given the pilot nature of this randomized study, a formal sample size calculation was not performed. The absence of prior empirical data precludes the estimation of an adequate sample size based on a predefined primary endpoint or an anticipated effect size between study arms. Instead, the sample size was determined pragmatically, with the primary aim of assessing feasibility and generating preliminary data to inform future adequately powered trials. Therefore, the sample size has been considered based on HGUGM Tumor Board patient registry with a total recruitment estimation of 30 HR-positive BC patients under 45 years old undergoing adjuvant treatment including OFS drugs expected to be maintained for at least 4 months at the time of study inclusion.

Data will be analyzed using Stata version 15 (StataCorp. 2017. *Stata Statistical Software: Release 15*. College Station, TX: StataCorp LLC) and SPSS Version 18 (IBM Corp. Released 2021. IBM SPSS Statistics for Windows, Version 29.0. Armonk, NY: IBM Corp).

Descriptive statistics will be generated for all clinical characteristics and outcome measures as appropriate.

The Kruskal-Wallis test for primary endpoints will assess between-groups range difference adjusted to initial values. The primary analysis will follow the intention-to-treat principle. If feasible, missing data will be addressed using appropriate imputation methods, such as multiple imputations. A multiple regression model will be performed to analyze the intervention program’s impact on body composition changes and weight. A nonparametric Kruskal-Wallis test for secondary endpoints and biomarker analysis will also evaluate between-arms range differences adjusted to initial values.

### Data management

Patient information will be handled in accordance with the Organic Law 3/2018, of December 5, on Personal Data Protection and guarantee of digital rights and the Regulation (EU) 2016/679 of the European Parliament and Council of 27^th^ April 2016 on Data Protection. Patient data will be handled pseudonymized, where included patients receive an alphanumeric code; only study investigators have access to patient identification and the final dataset.

HGUGM and Ejercicio y Cáncer center will conduct patient registration and codification, randomization, data collection, cleaning and monitoring electronically. No Data Monitoring Committee has been established due to the pilot nature of the study, but internal monitoring will ensure data quality. All data collected during the study will be securely stored and anonymized to prevent identification by third parties. PROs data will be collected directly from patients through electronic devices (smart-phones, tablets or personal computers). Individual records (informed consent, medical history records, laboratory data, etc.) will be securely maintained at HGUGM.

### Strengths and limitations of this study

This is a study with a comprehensive approach which integrates nutrition, exercise, and psycho-oncological support and provides a holistic intervention that addresses multiple aspects of patients’ health. The study’s individual care for each participant’s specific needs, would potentially enhance effectiveness and adherence to the intervention. The trial includes a wide range of primary, secondary, and exploratory outcomes and incorporates detailed measures, allowing for a thorough assessment of the intervention’s impact on various health parameters.

This study offers an opportunity to investigate cardiorespiratory and metabolic health in a BC patient population of increasing importance with long-term survival expectations, while also examining the influence of physiological characteristics and overall health status on responses to multimodal strategies incorporating exercise, nutrition, and psycho-oncological intervention.

To the best of our knowledge, this is the first study to investigate the feasibility and preliminary effects of an integrative approach combining individualized physical exercise, nutrition, and psycho-oncological support interventions in a highly specific subgroup of young breast cancer patients undergoing ovarian function suppression.

There are some limitations, such as a single-center design, which may limit the generalizability of the findings to other settings or populations. Moreover, the pragmatic pilot sample size may restrict the statistical power and robustness of the conclusions drawn from the study.

### Identification of potential sources of bias

Exercise programs trials for cancer patients can have biases that may affect their effectiveness and accessibility. Some potential biases that we identify are described below:

Exercise programs studies often target specific age and gender groups, potentially excluding younger or older patients who might benefit from tailored interventions and even leading to gender-specific findings that may not be universally applicable; furthermore, research frequently focuses on certain frequent cancer types, such as breast or prostate cancer, which may not address the needs of patients with less common cancers. Unfortunately, considering the nature of this study, designed to assess the potential benefits of a multimodal approach in a specific subgroup of BC patients, we acknowledge that sex, age, and gender biases are intrinsic to this research and, as such, are not subject to control.

Due to the limited ethnic and racial diversity in our country and community, this type of study may not adequately capture the heterogeneity of wider populations, which could restrict the external validity of the findings.

Addressing these biases will require more inclusive research approaches to ensure the generalizability and equitable benefit of exercise programs for all cancer patients.

## References

[1] Sociedad Española de Oncología Médica . Las cifras del cáncer en España (2025). Available online at: https://seom.org/images/LAS_CIFRAS_DMC2025.pdf (Accessed March 15, 2025).

[2] Francis PA Pagani O Fleming GF Walley BA Colleoni M Láng I . Tailoring adjuvant endocrine therapy for premenopausal breast cancer. New Engl J Med. (2018) 379:122–37. doi: 10.1056/NEJMoa1803164, PMID: 29863451 PMC6193457

[3] Francis PA Fleming GF Láng I Ciruelos EM Bonnefoi HR Bellet M . Adjuvant endocrine therapy in premenopausal breast cancer: 12-year results from SOFT. J Clin Oncol. (2023) 41:1370–5. doi: 10.1200/JCO.22.01065, PMID: 36493334 PMC10419521

[4] Gray RG Bradley R Braybrooke J Clarke M Hills RK Peto R . Effects of ovarian ablation or suppression on breast cancer recurrence and survival: Patient-level meta-analysis of 14,993 pre-menopausal women in 25 randomized trials. J Clin Oncol. (2023) 41:503–3. doi: 10.1200/JCO.2023.41.16_suppl.503

[5] Gradishar WJ Moran MS Abraham J Abramson V Aft R Agnese D . Breast cancer, version 3.2024, NCCN clinical practice guidelines in oncology. J Natl Compr Cancer Netw. (2024) 22:331–57. Available online at: https://jnccn.org/view/journals/jnccn/22/5/article-p331.xml (Accessed March 16, 2025)., PMID: 39019058 10.6004/jnccn.2024.0035

[6] Loibl S André F Bachelot T Barrios CH Bergh J Burstein HJ . Early breast cancer: ESMO Clinical Practice Guideline for diagnosis, treatment and follow-up. Ann Oncol. (2024) 35(2):159–82. doi: 10.1016/j.annonc.2023.11.016, PMID: 38101773

[7] Lu YS Wong A Kim HJ . Ovarian function suppression with luteinizing hormone-releasing hormone agonists for the treatment of hormone receptor-positive early breast cancer in premenopausal women. Front Oncol. (2021) 11:3299. doi: 10.3389/fonc.2021.700722, PMID: 34595110 PMC8477635

[8] Hojan K Molińska-Glura M Milecki P . Physical activity and body composition, body physique, and quality of life in premenopausal breast cancer patients during endocrine therapy–a feasibility study. Acta Oncol. (2023) 52:319–26. doi: 10.3109/0284186X.2012.744468, PMID: 23193959

[9] Kauffman RP Young C Castracane VD . Perils of prolonged ovarian suppression and hypoestrogenism in the treatment of breast cancer: Is the risk of treatment worse than the risk of recurrence? Mol Cell Endocrinol. (2021) 525:111181. doi: 10.1016/j.mce.2021.111181, PMID: 33529690

[10] Friedenreich CM Ryder-Burbidge C McNeil J . Physical activity, obesity and sedentary behavior in cancer etiology: epidemiologic evidence and biologic mechanisms. Mol Oncol. (2021) 15:790–800. doi: 10.1002/1878-0261.12772, PMID: 32741068 PMC7931121

[11] Demark-Wahnefried W Schmitz KH Alfano CM Bail JR Goodwin PJ Thomson CA . Weight management and physical activity throughout the cancer care continuum. CA Cancer J Clin. (2018) 68:64–89. doi: 10.3322/caac.21441, PMID: 29165798 PMC5766382

[12] Giugliano F Bertaut A Blanc J Martin AL Gaudin C Fournier M . Characteristics, treatment patterns and survival of patients with high-risk early hormone receptor-positive breast cancer in French real-world settings: an exploratory study of the CANTO cohort. ESMO Open. (2024) 9. Available online at: https://www.esmoopen.com/action/showFullText?pii=S2059702924017642., PMID: 39612621 10.1016/j.esmoop.2024.103994 PMC11647466

[13] Avgerinos KI Spyrou N Mantzoros CS Dalamaga M . Obesity and cancer risk: Emerging biological mechanisms and perspectives. Metabolism. (2019) 92:121–35. Available online at: http://www.metabolismjournal.com/article/S0026049518302324/fulltext., PMID: 30445141 10.1016/j.metabol.2018.11.001

[14] Iwase T Wang X Shrimanker TV Kolonin MG Ueno NT . Body composition and breast cancer risk and treatment: mechanisms and impact. Breast Cancer Res Treat. (2021) 186:273–83. doi: 10.1007/s10549-020-06092-5, PMID: 33475878

[15] Zieff GH Wagoner CW Paterson C Lassalle PP Lee JT . Cardiovascular consequences of skeletal muscle impairments in breast cancer. Sports (Basel). (2020) 8. doi: 10.3390/sports8060080, PMID: 32486406 PMC7353641

[16] Franceschi C Garagnani P Parini P Giuliani C Santoro A . Inflammaging: a new immune–metabolic viewpoint for age-related diseases. Nat Rev Endocrinol. (2018) 14:10. Available online at: https://www.nature.com/articles/s41574-018-0059-4., PMID: 30046148 10.1038/s41574-018-0059-4

[17] Michels N van Aart C Morisse J Mullee A Huybrechts I . Chronic inflammation towards cancer incidence: A systematic review and meta-analysis of epidemiological studies. Crit Rev Oncol Hematol. (2021) 157:103177. doi: 10.1016/j.critrevonc.2020.103177, PMID: 33264718

[18] Fiuza-Luces C Valenzuela PL Gálvez BG Ramírez M López-Soto A Simpson RJ . The effect of physical exercise on anticancer immunity. Nat Rev Immunol. (2024) 24:282–93. doi: 10.1038/s41577-023-00943-0, PMID: 37794239

[19] Campbell KL Winters-Stone KM Wiskemann J May AM Schwartz AL Courneya KS . Exercise guidelines for cancer survivors: consensus statement from international multidisciplinary roundtable. Med Sci Sports Exerc. (2023) 51:2375–90. doi: 10.1249/MSS.0000000000002116, PMID: 31626055 PMC8576825

[20] Shaikh H Bradhurst P Ma LX Tan SY Egger SJ Vardy JL . Body weight management in overweight and obese breast cancer survivors. Cochrane Database Syst Rev. (2020) 2020. doi: 10.1002/14651858.CD012110.pub2/full, PMID: 33305350 PMC8094215

[21] Demark-Wahnefried W Rogers LQ Gibson JT Harada S Frugé AD Oster RA . Randomized trial of weight loss in primary breast cancer: Impact on body composition, circulating biomarkers and tumor characteristics. Int J Cancer. (2020) 146:2784–96. doi: 10.1002/ijc.32637, PMID: 31442303 PMC7155016

[22] Schmidt T Van Mackelenbergh M Wesch D Mundhenke C . Physical activity influences the immune system of breast cancer patients. J Cancer Res Ther. (2017) 13(3):392–8. doi: 10.4103/0973-1482.150356, PMID: 28862198

[23] Koelwyn GJ Zhuang X Tammela T Schietinger A Jones LW . Exercise and immunometabolic regulation in cancer. Nat Metab. (2020) 2:849–57. doi: 10.1038/s42255-020-00277-4, PMID: 32929232 PMC9128397

[24] Ormel HL van der Schoot GGF Sluiter WJ Jalving M Gietema JA Walenkamp AME . Predictors of adherence to exercise interventions during and after cancer treatment: A systematic review. Psychooncology. (2018) 27:713. Available online at: /pmc/articles/PMC5887924/. 29247584 10.1002/pon.4612 PMC5887924

[25] Ranes M Wiestad TH Thormodsen I Arving C . Determinants of exercise adherence and maintenance for cancer survivors: Implementation of a community-based group exercise program. A qualitative feasibility study. PEC Innov. (2022) 1:100088. doi: 10.1016/j.pecinn.2022.100088, PMID: 37213720 PMC10194213

[26] American Thoracic Society American College of Chest Physicians . ATS/ACCP Statement on cardiopulmonary exercise testing. Am J Respir Crit Care Med. (2003) 167(2):211–77. doi: 10.1164/rccm.167.2.211, PMID: 12524257

[27] Fernandez C Firdous S Jehangir W Behm B Mehta Z Berger A . Cancer-related fatigue: perception of effort or task failure? Am J Hosp Palliat Care. (2020) 37(1):34–40. doi: 10.1177/1049909119849420, PMID: 31084200

[28] Michie S Ashford S Sniehotta FF Dombrowski SU Bishop A French DP . A refined taxonomy of behaviour change techniques to help people change their physical activity and healthy eating behaviours: The CALO-RE taxonomy. Psychol Health. (2011) 26:1479–98. doi: 10.1080/08870446.2010.540664, PMID: 21678185

[29] Ferguson B . ACSM’s guidelines for exercise testing and prescription 9th ed. 2014. J Can Chiropr Assoc. (2014) 58:328. Available online at: /pmc/articles/PMC4139760/.

[30] Ramos-Goñi JM Craig BM Oppe M Ramallo-Fariña Y Pinto-Prades JL Luo N . Handling data quality issues to estimate the spanish EQ-5D-5L value set using a hybrid interval regression approach. Value Health. (2018) 21:596–604. doi: 10.1016/j.jval.2017.10.023, PMID: 29753358

[31] Cella D Hernandez L Bonomi AE Corona M Vaquero M Shiomoto G . Spanish language translation and initial validation of the functional assessment of cancer therapy quality-of-life instrument. Med Care. (1998) 36:1407–18. doi: 10.1097/00005650-199809000-00012, PMID: 9749663

[32] Roman-Viñas B Serra-Majem L Hagströmer M Ribas-Barba L Sjöström M Segura-Cardona R . International Physical Activity Questionnaire: Reliability and validity in a Spanish population. European Journal of Sport Science. (2010) 10:297–304. doi: 10.1080/17461390903426667

[33] Morejón AJV García-Bóveda RJ Jiménez RVM . Escala de autoestima de Rosenberg: fiabilidad y validez en población clínica española. Apunt Psicol. 22:247–55. Available online at: https://www.apuntesdepsicologia.es/index.php/revista/article/view/53 (Accessd December 7, 2004).

[34] Herrero MJ Blanch J Peri JM De Pablo J Pintor L Bulbena A . A validation study of the hospital anxiety and depression scale (HADS) in a Spanish population. Gen Hosp Psychiatry. (2003) 25:277–83. doi: 10.1016/S0163-8343(03)00043-4, PMID: 12850660

[35] Ortega RM Requejo AM López-Sobaler AM . Registro de cosumo de alimentos y bebidas. Ortega RM Requejo AM , editors. Madrid: Manual de Nutrición Clínica (2015).

[36] Estruch R Ros E Salas-Salvadó J Covas MI Corella D Arós F . Primary prevention of cardiovascular disease with a mediterranean diet. Z fur Gefassmedizin. (2013) 10:28. doi: 10.1056/NEJMoa1200303, PMID: 23432189

[37] Shams-White MM Brockton NT Mitrou P Romaguera D Brown S Bender A . Operationalizing the 2018 world cancer research fund/american institute for cancer research (WCRF/AICR) cancer prevention recommendations: A standardized scoring system. Nutrients. (2019) 11. doi: 10.3390/nu11071572, PMID: 31336836 PMC6682977

[38] Lope V Guerrero-Zotano A Ruiz-Moreno E Bermejo B Antolín S Montaño Á . Clinical and sociodemographic determinants of adherence to world cancer research fund/american institute for cancer research (WCRF/AICR) recommendations in breast cancer survivors-health-epiGEICAM study. Cancers (Basel). (2022) 14. doi: 10.3390/cancers14194705, PMID: 36230628 PMC9561971

[39] Sandín B Chorot P Lostao L Joiner TE Santed MA Valiente RM . Escalas PANAS de afecto positivo y negativo: Validacion factorial y convergencia transcultural. Psicothema. (1999) 11. Available online at: https://www.kerwa.ucr.ac.cr/server/api/core/bitstreams/b095be83-ced8-47d1-9db1-525b5747f7b5/content (Accessed November 18, 2024).

[40] Leyton M Batista M Lobato S Jiménez Y Ayudante P Universidad D . Validación del cuestionario del modelo transteo?rico del cambio de ejercicio físico. Rev Internacional Med y Cienc la Actividad Física y del Deporte. 19:329–50. Available online at: https://revistas.uam.es/rimcafd/article/view/rimcafd2019.74.010.

[41] Díaz Fonte J Cruzado JA Díaz Fonte J Cruzado JA . El Modelo Transteórico y el ejercicio en supervivientes de cáncer de mama. Clin Salud. (2021) 32:129–37. Available online at: https://scielo.isciii.es/scielo.php?script=sci_arttext&pid=S1130-52742021000300129&lng=es&nrm=iso&tlng=es.

[42] Requena GC Salamero M Gil F . Validación del cuestionario MOS-SSS de apoyo social en pacientes con cáncer. Med Clin (Barc). (2007) 128:687–91. doi: 10.1157/13102357, PMID: 17540143

[43] Priede A Andreu-Vaillo Y Martínez López P Ruíz Torres M Hoyuela F González Blanch C . Validación de la Escala MOS-SSS de apoyo social en una muestra de pacientes oncológicos recién diagnosticados. Proc Int Congress Clin Psycol. (2016), 45–53.

[44] Hojman P Dethlefsen C Brandt C Hansen J Pedersen L Pedersen BK . Exercise-induced muscle-derived cytokines inhibit mammary cancer cell growth. Am J Physiol Endocrinol Metab. (2011) 301. doi: 10.1152/ajpendo.00520.2010, PMID: 21653222

