# Supplementary material for: Randomized pilot study of an individualized multimodal exercise, nutrition, and behavior intervention in breast cancer patients treated with ovarian function suppression: protocol proposal for The OvS Breast ENBI Project
Source: Front Oncol. 2025 Oct 27;15:1622622. doi: 10.3389/fonc.2025.1622622 (PMC12597807; doi:10.3389/fonc.2025.1622622)
Supplement: Supplementary file 1 [file SupplementaryFile1.docx]

Supplementary Appendix 1: Standard Protocol Items Recommendations for Interventional Trials (SPIRIT) checklist.

**Administrative information**

1. **Title:** Randomized pilot study of individualized multimodal exercise, nutrition, and behavior intervention in breast cancer patients treated with ovarian function suppression: Protocol proposal for The OvS Breast ENBI Project (ENBI).
2. **Trial registration**

**2a)** clinicaltrials.gov NCT06727487

**2b)** World Health Organization Trial Registration Data Set

## Primary Registry and Trial Identifying Number: clinicaltrials.gov NCT06727487; OvS Breast: Ovarian Suppression in Breast Cancer Interventions (ENBI)

1. **Date of Registration in Primary Registry**: 2024-12-11
2. **Secondary Identifying Numbers**: ENBI_2023 (sponsor), ENBI (HGUGM ethics committee)
3. **Source(s) of Monetary or Material Support:** None
4. **Primary sponsor:** Universidad Pontificia Comillas.
5. **Secondary Sponsor(s)**: None.
6. **Contact for Public Queries**

Blanca Herrero López, MD. Email: [b.herrero88@gmail.com](mailto:b.herrero88@gmail.com); Phone number: 914269823; Postal Address: Hospital General Universitario Gregorio Marañón Centro Oncológico, Calle Maiquez, 7, 28007, Madrid (España). Recruiting.

Soraya Casla Barrio, MSc, Ph.D. Email: [Soraya.casla@ejercicioycancer.es](mailto:Soraya.casla@ejercicioycancer.es); Phone: +34 686682364; Address: Ejercicio y Cáncer Center. Plaza Niño Jesús, 5. 28009 Madrid (Spain). Intervention coordinator.

1. **Contact for Scientific Queries**

Blanca Herrero López, MD. Email: [b.herrero88@gmail.com](mailto:b.herrero88@gmail.com); Phone number: 914269823; Postal Adress: Hospital General Universitario Gregorio Marañón Centro Oncológico, Calle/Maiquez, 7, 28007, Madrid (España). Recruiting.

Soraya Casla Barrio, MSc, Ph.D. Email: [Soraya.casla@ejercicioycancer.es](mailto:Soraya.casla@ejercicioycancer.es); Phone: +34 686682364; Adress: Ejercicio y Cáncer Center. Plaza Niño Jesús, 5. 28009 Madrid (Spain). Intervention coordinator.

Julio de la Torre, RN, MsN, Ph.D. Email: [juliodelatorre@comillas.edu](mailto:juliodelatorre@comillas.edu) Phone: +34678581021 Universidad Pontificia Comillas. Health Sciences Department. Avenida San Juan de Dios, 1. Ciempozuelos. 28350. Madrid. Spain. Study Coordinator.

1. **Public Title**: OvS Breast: ENBI Project (Ovarian Suppression in Breast Cancer: Exercise, Nutrition and Behavioural Intervention Project)**.**
2. **Scientific Title**: OvS Breast: ENBI Project (Ovarian Suppression in Breast Cancer: Exercise, Nutrition and Behavioural Intervention Project).
3. **Countries of recruitment:** Spain.
4. **Health Condition(s) or Problem(s) Studied**: Premenopausal breast cancer patients undergoing adjuvant ovarian suppression treatment.
5. **Intervention(s):** See SPIRIT checklist section 11a-c

Intervention Arm: 12-weeks individualized nutritional, exercise, and psycho-oncological intervention on body composition and weight.

Control Arm: World Health Organization (WHO) basic recommendations on healthy lifestyle habits

1. **Key Inclusion and Exclusion Criteria:** Eligible patients should meet the following inclusion and exclusion criteria:

Inclusion criteria

- At least 18 and up to 45 years of age at the time of consent.
- Histologically confirmed HR-positive stage I to III invasive BC.
- Premenopausal status clinically defined as a patient who maintains menstruation prior to chemotherapy initiation if his has taken place and/or regular menstruation at the time of consent.
- Completed locoregional treatment (surgery and radiotherapy).
- Ongoing adjuvant treatment including OFS drugs expected to be maintained for at least 4 months at the time of consent.
- Functional status by the Eastern Cooperative Oncology Group (ECOG) scale 0-1.
- Ability to understand and give informed consent (IC).

Exclusion criteria

- Any medical contraindication to exercise practice.
- Any American Thoracic Society (ATS) contraindications for cardiopulmonary exercise testing^1^ .
- Active metastatic BC or other concurrent cancer diagnosis at the time of consent.
- Pregnant or breastfeeding women.
- Alcohol or other drugs abuse (excluding smoking) defined as a pattern of usual consumption that results in physical, mental or social functioning impairment.
- Any condition that makes the patient ineligible based on the investigator’s criteria.

1. **Study Type**
   - Type of study: Interventional study.
   - Study design including:
     - Method of allocation: Randomized study.
     - Masking: open-label study.
     - Assignment: two arms, crossover assignment allowed according to patient choice after the 2^nd^ follow-up time point.
     - Purpose: Treatment
   - Phase (if applicable): pilot study.
2. **Date of first enrollment:** August 2024
3. **Sample Size**

The trial was planned to enroll 30 patients.

Finally, participants enrolled were 27: 3. Patients did retire informed consent after being enrolled.

1. **Recruitment status:** Complete.
2. **Primary Outcome(s):** See section 12 SPIRIT checklist below.

Impact of the combined intervention on weight and body composition: This endpoint will be assessed by the following variables: weight, height, body mass index (IMC), waist and hip circumferences, waist-to-hip ratio (WHR), fat mass, lean mass and extracellular water which will be obtained with Tanita BC-601 Gold weight scale and bioelectrical impedance analysis as well as a measuring tape.

Timepoints of primary interest: Primary outcomes will be assessed immediately before the 12-week intervention period and at the end of it.

1. **Key Secondary Outcomes:** See section 12 SPIRIT checklist below.
   - The name of the outcome, the metric or method of measurement used: evaluating the impact of combined intervention on 1) CRF estimated by Bruce test and maximum oxygen uptake (VO2max), 2) cardiac variability assessed by resting and maximal heart rate (HR), 3) endurance performance measured by capillary lactate levels at rest, maximum effort and two minutes after maximum effort, 4) muscle strength evaluated by upper and lower body dynamometry, 5) physical function assessed by 30-second sit-to-stand test and 6-minute walking test, and 6) potentially modifiable laboratory parameters such as complete blood count, glucose levels, complete liver and renal function panels, electrolyte concentrations, protein and albumin levels, complete lipid profile and thyroid function. Also, patient reported outcomes (PROs) are considered secondary endpoints (1) EuroQoL-5D^2^ (quality of life (QoL)), 2) FACIT-Fatigue^3^ (fatigue), 3) International Physical Activity Questionnaire^4^ (IPAQ) (physical activity level), 4) Rosenberg self-esteem scale^5^ (self-esteem), 5) Hospital Anxiety and Depression Scale^6^ (HADS) (anxiety and depression levels)). Concerning systemic therapy-associated adverse events, investigators will evaluate and grade them according to CTCAE version 5 as secondary endpoints (lymphedema, peripheral neuropathy, arthralgias, irritability, insomnia, vaginal dryness, decreased libido, hot flashes). Nutritional and psychological status are also included as secondary endpoints. A 3-day nutritional record will assess nutritional status, PREDIMED Mediterranean Diet Adherence Score^7,^ and compliance with WCRF/AICR cancer prevention recommendations^8,9^. Positive and negative affect scale (PANAS)^10^, the transtheoretical model of physical activity change questionnaire11,12, and the MOS social support survey^13,14^ will serve to evaluate psychological status.
   - The timepoint(s) of interest: Secondary endpoints will be assessed immediately before the 12-week intervention period and at the end of it. They will also be evaluated at 6- and 12-month follow-up timepoints.
2. **Ethics Review**
   - Approved.
   - Date of approval: 6th October 2023
   - Hospital General Universitario Gregorio Marañón Ethics Committee.

HGUGM Ethics Committee contact information:

Santos Puerta Cruz

**Secretaría Técnica CEIm**

Fundación para la Investigación Biomédica. Hospital Gregorio Marañón. Pabellón de Gobierno 1ª Planta

C/ Dr. Esquerdo, 46, 28007 MADRID

Phone number: +34 91 586 7007

2nd Phone number: +34 91 426 93 78.

Email address: [ceim.hgugm@salud.madrid.org](mailto:ceim.hgugm@salud.madrid.org)

1. **Completion date**: Study completion is planned for April 2026 (last follow-up time point).
2. **Summary Results**: There are no results posted for this study yet.
3. **IPD sharing statement**
   Plan to share IPD: Yes. (Yes, No, Undecided)

Plan description: A complete publication plan will be considered, covering everything from the protocol to disseminating the results in scientific journals and scientific forums, such as specialized conferences.

Time frame: 2 years from the last patient is included.

IPD Sharing supporting information type: Study Protocol, Statistical Analysis Plan (SAP), Informed Consent Form (ICF), Clinical Study Report (CSR), Analytic Code.

1. **Date and version identifier**

Issue date: 05 Oct 2023

Protocol amendment number: 5. Since the Hospital General Universitario Gregorio Marañón Ethics Committee protocol approval, no amendment has been made.

Authors: Blanca Herrero López; María del Monte Millán

1. **Funding**

The present work is an academic trial developed by several coordinated institutions (Hospital General Universitario Gregorio Marañón, Ejercicio y Cáncer, Universidad Pontificia Comillas): The design, management, analysis and reporting of the study are entirely independent of the financial grant obtained from Daiichi Sankyo. Daiichi Sankyo grant will cover external services hiring (statistical analysis, biomarker analysis, courier service), fungible goods, as well as travel and publication expenses.

1. **Roles and responsibilities**

**5a)** **Names, affiliation and roles of protocol contributors:** Blanca Herrero López^1^, Mónica Castellanos Montealegre^2^, Candelaria Soulas^2^, Begoña Arbulo Rufrancos^3^, María Luisa García-Ontiveros Cuéllar^3^, María del Monte Millán^4^, Lucía Villarejo López^4^, Sara López-Tarruella Cobo^1^, Yolanda Jerez Gilarranz^1^, Isabel Echavarría Díaz-Guardamino^1^, Pablo Jara^1^, Miguel Martín Jiménez^1^, Tatiana Massarrah Sánchez^4^, María Jesús Martínez Beltrán^5^, Julio César de la Torre Montero^5*^ & Soraya Casla Barrio^2*^.

^1^ Department of Medical Oncology, Breast Cancer Unit, Hospital General Universitario Gregorio Marañón, Madrid, Spain.

^2^ Centro Ejercicio y Cáncer, Madrid, Spain.

^3^ Department of Psychiatry and Clinical Psychology, Hospital General Universitario Gregorio Marañón, Madrid.

^4^ Department of Medical Oncology, Translational Research Unit, Hospital General Universitario Gregorio Marañón, Madrid, Spain.

^5^ San Juan de Dios Nursing and Physiotherapy School, Universidad Pontificia Comillas, Ciempozuelos, Madrid, Spain.

BHL is the principal investigator. BHL, SCB, MCM and JCDTM were involved in conception of the study. BHL, SCB, MCM, CS, MGOC, BAR, MMM, SLTC, MMJ, JCDTM and MJMB were involved in developing the study design. SCB and MJMB designed statistical analysis. BHL, SCB, MCM, MMM and LVL developed the data management plan. BHL, SLTC, YJG, IEDG and PJ are recruiting patients. MMM, LVL and TMS play a primary role on organizing patients processes in the study. SCB and MCM are responsible for designing and developing exercise training sessions. CS carries out nutritional sessions. BAR and MGOC lead psycho-oncological support sessions. BHL wrote the first draft of the manuscript.

5b) **Name and contact information for the trial sponsor:** Universidad Pontificia Comillas & San Juan de Dios Foundation. Avenue San Juan de Dios,1, 28350 Ciempozuelos, Madrid, Spain.

Contact Name: Julio César de la Torre Montero.

Phone number +34 918 933 769.

Email: [juliodelatorre@comillas.edu](mailto:juliodelatorre@comillas.edu)

**5c) Role of study sponsor and funders in study design:** Neither the sponsor nor the partial funder company has any role in study design and will not have any role during its execution, analyses, interpretation of the data, or decision to submit results.

**5d) Composition, roles and responsibilities of the coordinating centre, steering committee, endpoint adjudication committee, data management team, etc.**

See section 5a. Coordinating Centre is Hospital General Universitario Gregorio Marañón (HGUGM), which has been involved in the conception of the study and developing the study design, data management plan, patient recruitment, and processes organization, along with psycho-oncological support sessions. The Exercise and Cancer Center is involved in designing and developing exercise training sessions as well as nutritional sessions. HGUGM and the Exercise and Cancer Centre are involved in data collection.

**Introduction**

1. **Background and rationale**

**6a) Description of research question and justification for undertaking the trial:**

Introduction: Breast cancer (BC) is the most frequent and incident cancer in Spanish women. Hormone receptor (HR) - positive BC is the most common subtype, accounting for approximately 75% of BC cases^15^.

Ovarian function suppression (OFS) combined with endocrine therapy (gonadotropin-releasing hormone agonists (GnRHa) administered along with tamoxifen (TAM) or aromatase inhibitors (AI)) have demonstrated a benefit in terms of relapse-free survival (RFS) and overall survival (OS) in premenopausal women diagnosed with HR-positive BC; this benefit is mainly focused on patients diagnosed at younger age, particularly those harboring tumors with other high risk clinicopathological features^16–18^. International clinical practice guidelines recommend OFS strategy for these scenarios ^19,20^.

Mechanisms: However, relevant cardiorespiratory, metabolic, sexual, as well as emotional adverse events have been associated with these treatments ^16,21^: Unfavorable body composition changes such as fat mass and weight increases, that can be observed from the first months of treatment^22,23^, are directly associated to higher BC relapse risk^24,25^ along with a potential loss of adherence to endocrine treatment^26^.

Body fat increments along with muscle mass loss result in a proinflammatory^27^ and dysfunctional immune system which has been linked to cardiovascular disease development and poor outcomes in cancer patients^28–30^.

Existing knowledge: Scientific evidence suggests that multimodal, individualized interventions combining nutrition, exercise and psycho-oncological support tailored to physiological needs, baseline physical condition and potential adverse events are safe^31^. This integrative approach has been shown to be effective in achieving a healthy weight and body composition in BC patients^32,33^. Additionally, this strategy may contribute to immune system functionality restoration^34,35^ and enhance cardiorespiratory fitness (CRF) leading to a positive impact on quality of life (QoL)^32,35^.

Need for a trial: Unfortunately, adherence to these combined programs have been found to be rather low. Psycho-oncological support and behavioral interventions would be able to stimulate lifestyle changes in BC patients and improve adherence rates^36,37^. Despite the plethora of evidence, there is no scientific consensus on the optimal exercise dose (intensity, duration and frequency) or the specific type of nutritional intervention that should be applied in premenopausal BC patients undergoing OFS treatment. Therefore, we consider that these interventions should be individualized and tailored to patients´ specific characteristics and factors influencing adherence. The present study has been designed to evaluate the impact of an individualized exercise and nutritional intervention combined with psycho-oncological support in premenopausal BC patients receiving adjuvant OFS treatment.

**6b) Explanation for choice of comparators:**

Nowadays standard practice does not include specific exercise, nutrition and/or psycho-oncological support for BC patients undergoing OFS adjuvant treatment; these patients only receive recommendations on healthy lifestyle habits. This is the reason why the intervention arm of this study consists of a 12-week individualized nutritional, exercise, and psycho-oncological support intervention, while control arm patients will only receive the World Health Organization (WHO) basic recommendations on healthy lifestyle habits. However, considering the expected benefits for patients allocated to the intervention arm, crossover is allowed for patients in the control group by the end of the last follow-up analysis according to patient choice.

1. **Specific objectives and hypotheses:**

Research hypothesis: Multimodal individualized intervention combining nutrition, exercise, and psycho-oncological support is a better strategy for controlling cardiovascular, metabolic, sexual, and emotional adverse events associated with OFS adjuvant therapy in premenopausal BC patients compared to receiving the World Health Organization (WHO) basic recommendations on healthy lifestyle habits.

Primary objective: to evaluate the impact of the combined and individualized intervention on weight reduction and favorable body composition modifications.

Secondary objectives: the secondary objectives aim to evaluate the impact of the combined intervention program on several variables related to exercise intervention, nutritional and psychological status, along with the treatments´ side effects.

Exploratory objectives: To evaluate the potential impact of the multimodal and individualized intervention on pro-inflammatory markers (C-reactive protein (CRP), tumor necrosis factor alpha (TNF-α)), anti-inflammatory markers (adiponectin^27^) and oncostatin-M (multifunctional myokine that has been related to apoptosis simulation in BC cell lines^38^).

1. **Description of trial design:** The ENBI trial is designed as a unicentric, open-label, 2:1 randomized pilot study with two parallel groups and permitted cross-over for patients in the control group when the last follow-up analysis has taken place. The primary endpoint is to evaluate the impact of a 12-week individualized nutritional, exercise, and psycho-oncological intervention on body composition and weight (intervention group) compared to receiving the World Health Organization (WHO) basic recommendations on healthy lifestyle habits in premenopausal HR-positive BC patients undergoing adjuvant OFS treatment (control group).

**Methods: Participants, interventions and outcomes**

1. **Study Setting: Description of study settings.** As a pilot study, ENBI has been developed as a unicentric trial: we chose HGUGM in Madrid (Spain) as the recruitment centre and the place where psycho-oncological intervention is developed in collaboration with the Exercise and Cancer Centre for exercise and nutritional interventions.
2. **Eligibility criteria: Inclusion and exclusion criteria for participants:** Patients (or a representative) must provide written, informed consent before any study procedures occur.

Eligible patients should meet the following inclusion and exclusion criteria:

Inclusion criteria

- At least 18 and up to 45 years of age at the time of consent.
- Histologically confirmed HR-positive stage I to III invasive BC.
- Premenopausal status is clinically defined as a patient who maintains menstruation prior to chemotherapy initiation if he has taken place and/or regular menstruation at the time of consent.
- Completed locoregional treatment (surgery and radiotherapy).
- Ongoing adjuvant treatment, including OFS drugs, is expected to be maintained for at least 4 months at the time of consent.
- Functional status by the Eastern Cooperative Oncology Group (ECOG) scale 0-1.
- Ability to understand and give informed consent (IC).

Exclusion criteria

- Any medical contraindication to exercise practice.
- Any American Thoracic Society (ATS) contraindications for cardiopulmonary exercise testing^1^ .
- Active metastatic BC or other concurrent cancer diagnosis at the time of consent.
- Pregnant or breastfeeding women.
- Alcohol or other drug abuse (excluding smoking) is defined as a pattern of usual consumption that results in physical, mental, or social functioning impairment.
- Any condition that makes the patient ineligible is based on the investigator’s criteria.

**Individuals performing interventions:** exercise training sessions will be guided by an exercise physiologist specialized in oncology, a cancer expert nutritionist will perform nutritional intervention sessions, and psycho-oncological support sessions will be carried out by a psycho-oncology specialist.

1. **Interventions**

**11a-c) Interventions for each group/criteria for discontinuing or modifying allocated interventions/strategies to improve adherence.**

Physical exercise intervention

- Materials: Scheduled exercise sessions at a cancer exercise centre led by two exercise physiologists specializing in cancer patients.
  - Aerobic/cardiovascular exercise: step, walking, and heart rate-controlled running.
  - Strength and neuromotor exercise: rubber bands, free weights.
- Methods:
  - Professionals: Two exercise physiologists specialized in cancer patients will carry out the intervention; they harbor a PhD on this topic and a bachelor’s degree on Physical Activity and Sport Sciences.
  - Location: Exercise and Cancer Centre; a cancer-specialized exercise centre. Outdoors activities will be developed at El Retiro park (Madrid).
  - Procedure: Supervised in-person exercise sessions combined with at-home exercises from the fifth session onwards.
  - Type: Cardiovascular exercise for 15-50 min/session; strength and neuromotor exercise for at least 20 min/session; stretching for 10 min/session.
  - Intensity:
    - Cardiovascular exercise: patients will work at 55-100% of Heart Rate Reserve (HRR); it will be measured with a heart rate monitor and exercise intensity will be assessed through Borg perception of effort scale.
    - Strength exercise: patients will work at cumulative intensity assessed by total kilograms (kg) moved per session. It will be collected as kg moved in each session and intensity will be assessed through Borg perception of effort scale.
    - Neuromotor exercise execution will increase from 40 seconds up to 1.5 minutes.
  - Frequency: two sessions per week.
  - Session duration: 55-75 minutes.
  - Intervention duration: 12 weeks.
  - Planned adaptations:
    - Resting time modifications according to each patient baseline physical condition.
    - Exercise adjustments based on previous or concurrent arthralgias and/or myalgias.
    - Impact reduction depending on possible pelvic floor problems.
    - Exercise intensity adjustments according to recovery from the previous session.
  - Adherence: the following tools are intended to promote patient adherence to the program:
    - Exercise supervision by exercise professionals with specific training in cancer patients.
    - Home adherence monitoring using the free Polar Beat smartphone app available for Android and iOS.
    - Attendance control in face-to-face sessions.
    - Adverse events monitoring as well as other eventualities that could potentially interfere with workout development e.g. muscle injuries, extreme temperatures, etc.)

Nutritional intervention

- Materials: Individual sessions with a cancer expert nutritionist. The following materials will be used:
  - 3-day nutritional record.
  - Predimed Questionnaire.
  - Compliance with WCRF/AICR recommendations scale.
  - Daily food questionnaire
- Methods:
  - Professionals: A cancer expert nutritionist will perform nutritional intervention.
  - Location: Exercise and Cancer Centre and, if necessary, online sessions.
  - Procedure: Before and after nutritional intervention a nutritional status assessment will be carried out with the materials described above.
  - Frequency: 3 nutrition sessions (week 4,10 and 16).
  - Session duration: 35-45 minutes per session.
  - Intervention duration: 12 weeks.
  - Planned adaptations:
    - Recommendations for adjustments based on food allergies or intolerances.
    - Recommendations will be adapted based on diet-related diseases (e.g. cardiovascular risk factors), digestive tract diseases o symptoms (e.g. inflammatory bowel disease, residual treatment-related adverse events, etc.).
    - Recommendations adapted to vegan or vegetarian diet.
  - Adherence: The following tools are intended to promote patient adherence to the program:
    - Program delivered by a cancer expert nutritionist.
    - Patients will receive standardized as well as individualized nutritional intervention guidelines.
    - Daily log monitoring will be useful to determine if nutrition lifestyle changes are taking place.
    - Nutritional recommendations could be modified in case any adverse event related to them is seen.

Psycho-oncological intervention

- Materials: Three face-to-face group sessions with a cancer expert psychologist. The following questionnaires will be used:
  - Positive and negative effects on the scale (PANAS).
  - Transtheoretical model of physical activity change questionnaire.
  - MOS social support survey.
- Methods:
  - Professionals: Two clinical psychologists specialized in cancer patients.
  - Location: Hospital General Universitario Gregorio Marañón. Oncology Centre.
  - Procedure: Three face-to-face group sessions: an initial intervention session with psychological status assessments, an intermediate session to monitor psychological status evolution and a final session to evaluate achievements and their impact on mood and future projection.
  - Frequency: 3 nutrition sessions (week 4,10 and 16).
  - Session duration: 90 minutes per session.
  - Intervention duration: 12 weeks.
  - Planned adaptations:
    - Language adaptations and examples based on patients’ needs and previous repertoire.
  - Adherence: the following tools are intended to promote patient adherence to the program:
    - Group participation stimulation by shared goals identification.
    - Working on a security, respect and confidentiality place.
    - Encourage patient self-observation and evaluation.

**11d) Relevant concomitant care and interventions that are permitted or prohibited during the trial:** There is neither specific concomitant care/interventions permitted nor prohibited during the trial.

1. **Outcomes**

The primary endpoint is to evaluate the impact of combined intervention on weight and body composition. This endpoint will be assessed by the following variables: weight, height, body mass index (IMC), waist and hip circumferences, waist-to-hip ratio (WHR), fat mass, lean mass and extracellular water which will be obtained with Tanita BC-601 Gold weight scale and bioelectrical impedance analysis as well as a measuring tape.

The secondary endpoints include evaluating the impact of combined intervention on 1) CRF estimated by Bruce test and maximum oxygen uptake (VO2max), 2) cardiac variability assessed by resting and maximal heart rate (HR), 3) endurance performance measured by capillary lactate levels at rest, maximum effort and two minutes after maximum effort, 4) muscle strength evaluated by upper and lower body dynamometry, 5) physical function assessed by 30-second sit-to-stand test and 6-minute walking test, and 6) potentially modifiable laboratory parameters such as complete blood count, glucose levels, complete liver and renal function panels, electrolyte concentrations, protein and albumin levels, complete lipid profile and thyroid function.

Patient reported outcomes (PROs) are also considered secondary endpoints. They will be assessed and collected using validated questionnaires: 1) EuroQoL-5D^2^ (quality of life (QoL)), 2) FACIT-Fatigue^3^ (fatigue), 3) International Physical Activity Questionnaire^4^ (IPAQ) (physical activity level), 4) Rosenberg self-esteem scale^5^ (self-esteem), 5) Hospital Anxiety and Depression Scale^6^ (HADS) (anxiety and depression levels).

Concerning systemic therapy-associated adverse events, investigators will evaluate and grade them according to CTCAE version 5 as secondary endpoints (lymphedema, peripheral neuropathy, arthralgias, irritability, insomnia, vaginal dryness, decreased libido, hot flashes).

Finally, nutritional and psychological status are also included as secondary endpoints. Nutritional status will be assessed by a 3-day nutritional record, PREDIMED Mediterranean Diet Adherence Score^7^ and compliance with WCRF/AICR cancer prevention recommendations^8,9^. Positive and negative affect scale (PANAS)^10^, transtheoretical model of physical activity change questionnaire^11,12^ and MOS social support survey^13,14^ will serve to evaluate psychological status.

Primary and secondary endpoints will be assessed immediately before the 12-week intervention period and at the end of it. They will be also evaluated at 6- and 12-months follow up timepoints.

Exploratory endpoints: Chronic inflammation has been linked to cancer development^39^. On the other hand, skeletal muscle acts as an endocrine organ whose contraction releases myokines (cytokines or peptides); exercise-induced myokines can exert an anti-inflammatory action^40^. The biomarker test proposed for this study aims to evaluate the potential impact of the combined intervention on pro-inflammatory markers (C-reactive protein (CRP), tumor necrosis factor alpha (TNF-α)), anti-inflammatory markers (adiponectin^27^) and oncostatin-M (multifunctional myokine that has been related to apoptosis simulation in BC cell lines^38^).

Exploratory endpoints will be assessed immediately before the 12-week intervention period and at the end of it.

1. **Participant timeline**

The following figure summarizes the study assessments from enrolment. We define week 1 to 3 as the moment in which patients are enrolled at HGUGM BC unit and we collect demographic information. Initial and final assessments take place the week before and after the 12-week intervention period (week 4 and 17), respectively. Blood for secondary endpoints and biomarker analysis is collected on week 4 and 17. On week 40 and 64, 6 and 12 months after the 12-week intervention respectively, a new general assessment (physical, nutritional and psycho-oncological evaluation) along with blood collection for secondary endpoints take place.


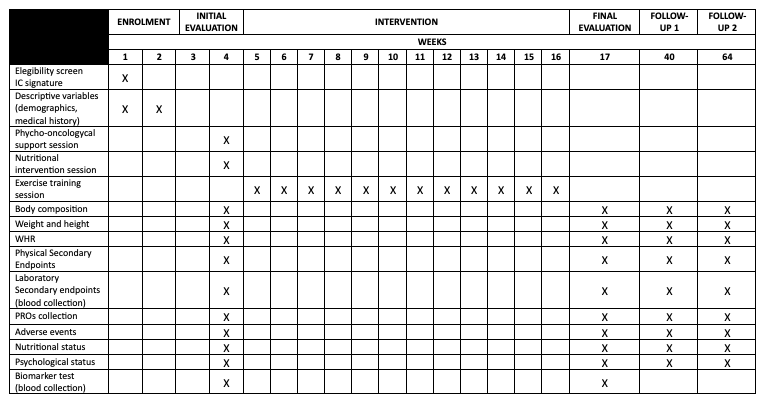


1. **Sample size**

No previous data allow investigators to establish an estimated sample size according to a pre-established main objective expected difference between the two study arms. Therefore, this trial has been designed as a pilot study where the sample size has been considered based on HGUGM Tumor Board patient registry with a total recruitment estimation of 30 HR-positive BC patients under 45 years old undergoing adjuvant treatment including OFS drugs expected to be maintained for at least 4 months at the time of study inclusion.

1. **Recruitment: strategies for achieving adequate participant enrollment.** Eligible patients will be identified from the HGUGM Tumor Board patient registry. Afterwards, physicians at HGUGM BC Unit will specifically make a phone call to those patients who will be offered to attend an appointment with principal investigator (PI). This appointment will serve to specifically explain trial hypothesis, duration, procedures and objectives, as well as potential benefits derived from their participation in the trial; furthermore, patients will be able to ask the PI any question they have about the study and will be offered to specifically talk to exercise experts, psychologists and nutritionists delivering trial intervention if they wish. Patients will provide written informed consent if they are willing to be included in the trial after the PI appointment has taken place.

**Methods: Assignment of interventions (for controlled trials)**

**16a. Allocation: Sequence generation**

The randomization process was centrally performed with a 2:1 ratio to intervention and control groups.

The allocation sequence will be generated using computer-based random number generation. No stratification factors are planned due to the pilot nature of the study.

**16b. Allocation concealment mechanism**

The allocation sequence will be concealed using a centralized assignment system, and the allocation will not be revealed to participants until enrollment is confirmed.

**16c. Implementation**

The allocation sequence will be generated by a member of the research team not involved in recruitment. Clinical investigators at HGUGM will enroll participants and assign them to study arms.

**Blinding (masking)**

**17a. Who will be blinded**

This is an open-label study due to the nature of the intervention. Patients, investigators, and clinical research coordinators will be aware of the allocation. No blinding is applied.

**17b. Unblinding procedure**

Not applicable, as the study is not blinded.

**Methods: Data collection, management, and analysis**

**18a. Data collection methods**

Baseline, outcome, and follow-up data are collected through validated questionnaires, physical assessments, laboratory tests, and biomarker analyses.
Data quality is promoted through validated instruments and pseudonymization. Assessment tools include EuroQoL-5D, FACIT-Fatigue, IPAQ, HADS, PANAS, etc.; their reliability/validity is referenced.

**18b. Participant retention and follow-up**

Follow-up assessments are scheduled at the end of the 12-week intervention and 6 and 12 months. To promote retention, regular contact and reminders will be used. If a participant discontinues the intervention, efforts will be made to collect primary outcome data at scheduled time points.

**19. Data management**

Patient data are pseudonymized using alphanumeric codes. HGUGM and Ejercicio y Cancer conduct data collection, entry, cleaning, and monitoring. PROs are collected electronically. The protocol refers to compliance with Spanish data protection law and EU GDPR.

**20a. Statistical methods for main outcomes**

Descriptive statistics will be used. Kruskal-Wallis tests and multiple regression models will assess the impact of the intervention on weight and body composition.
Software: Stata v15 and SPSS v29.

**20b. Additional analyses**

The Nonparametric Kruskal-Wallis test will also be used for secondary endpoints and biomarker analysis. No mention of subgroup or adjusted analyses.

**20c. Analysis of population and handling of missing data**

The primary analysis will follow the intention-to-treat principle. If feasible, missing data will be addressed using appropriate imputation methods, such as multiple imputation.

**Methods: Monitoring**

**21a. Data Monitoring Committee (DMC)**

Due to the pilot nature and low risk of the intervention, a data monitoring committee has not been established.

**21b. Interim analyses and stopping guidelines**

No interim analyses or formal stopping rules are planned for this pilot study.

**22. Harms**

Adverse events will be evaluated and graded using CTCAE v5.0. The study assesses a broad set of therapy-associated adverse events.

**23. Auditing**

No independent auditing is planned. Data quality will be internally monitored by study coordinators.

**Ethics and dissemination**

**24. Research ethics approval**

Approved by the HGUGM Drug Research Ethics Committee (CEIm). Protocol complies with the Declaration of Helsinki.

1. **Protocol amendments**

Relevant protocol amendments will be communicated to investigators, the CEIm, participants (as needed), and updated in the clinical trial registry.

**26a. Informed consent**

Eligible patients provide written informed consent before any procedure.

**26b. Additional consent for ancillary studies**

No additional consent for ancillary studies involving biological specimens is currently planned.

1. **Confidentiality**

Personal data are pseudonymized. Only investigators have access to identifiable data. Compliance with Spanish and EU data protection laws.

1. **Declaration of interests**

All authors declare no competing financial or personal interests.

1. **Access to data**

Access to the final dataset will be restricted to study investigators. No contractual agreements limiting access are reported.

**30. Ancillary and post-trial care**

No specific provisions for post-trial care or compensation for trial-related harm are included, given the low-risk nature of the intervention.

**31a. Dissemination policy**

Results will be presented at scientific conferences and published in peer-reviewed journals.

**31b. Authorship eligibility**

Authorship will be decided by the PIs based on substantial contributions to study design, conduct, analysis, or manuscript writing, following ICMJE criteria.

**31c. Public access to data and protocol**

There are currently no plans to grant public access to the full protocol, participant-level dataset, or statistical code.

**Appendices**

1. **Informed consent materials**

Attach the model consent form as an appendix for submission.

1. **Biological specimens**

Blood samples will be collected to analyze inflammatory markers (CRP, TNF-α, adiponectin, oncostatin-M).

Storage and evaluation details are not fully described.
*Suggested text:* Biological specimens will be processed and stored according to standard operating procedures at HGUGM laboratories for biomarker analysis.

**BIBLIOGRAPHY**

1. ATS/ACCP Statement on cardiopulmonary exercise testing. Am J Respir Crit Care Med [Internet]. 2003 [cited 2023 May 27];167(2):211–77. Available from: https://pubmed.ncbi.nlm.nih.gov/12524257/

2. Ramos-Goñi JM, Craig BM, Oppe M, Ramallo-Fariña Y, Pinto-Prades JL, Luo N, et al. Handling Data Quality Issues to Estimate the Spanish EQ-5D-5L Value Set Using a Hybrid Interval Regression Approach. Value Health [Internet]. 2018 May 1 [cited 2023 Jun 17];21(5):596–604. Available from: https://pubmed.ncbi.nlm.nih.gov/29753358/

3. Cella D, Hernandez L, Bonomi AE, Corona M, Vaquero M, Shiomoto G, et al. Spanish language translation and initial validation of the functional assessment of cancer therapy quality-of-life instrument. Med Care [Internet]. 1998 [cited 2023 Jun 17];36(9):1407–18. Available from: https://pubmed.ncbi.nlm.nih.gov/9749663/

4. Roman-Viñas B, Serra-Majem L, Hagströmer M, Ribas-Barba L, Sjöström M, Segura-Cardona R. International Physical Activity Questionnaire: Reliability and validity in a Spanish population. https://doi.org/101080/17461390903426667 [Internet]. 2010 Sep [cited 2023 Jun 17];10(5):297–304. Available from: https://www.tandfonline.com/doi/abs/10.1080/17461390903426667

5. MOREJÓN AJV, GARCÍA-BÓVEDA RJ, JIMÉNEZ RVM. Escala de autoestima de Rosenberg: fiabilidad y validez en población clínica española. Apunt Psicol [Internet]. 2004 [cited 2023 Jun 17];22(2):247–55. Available from: https://www.apuntesdepsicologia.es/index.php/revista/article/view/53

6. Herrero MJ, Blanch J, Peri JM, De Pablo J, Pintor L, Bulbena A. A validation study of the hospital anxiety and depression scale (HADS) in a Spanish population. Gen Hosp Psychiatry [Internet]. 2003 [cited 2023 Jun 17];25(4):277–83. Available from: https://pubmed.ncbi.nlm.nih.gov/12850660/

7. Estruch R, Ros E, Salas-Salvadó J, Covas MI, Corella D, Arós F, et al. Primary prevention of cardiovascular disease with a mediterranean diet. Zeitschrift fur Gefassmedizin [Internet]. 2013 Apr 4 [cited 2023 Jun 19];10(2):28. Available from: https://www.nejm.org/doi/full/10.1056/NEJMoa1200303

8. Shams-White MM, Brockton NT, Mitrou P, Romaguera D, Brown S, Bender A, et al. Operationalizing the 2018 World Cancer Research Fund/American Institute for Cancer Research (WCRF/AICR) Cancer Prevention Recommendations: A Standardized Scoring System. Nutrients [Internet]. 2019 Jul 1 [cited 2023 Jun 17];11(7). Available from: https://pubmed.ncbi.nlm.nih.gov/31336836/

9. Lope V, Guerrero-Zotano A, Ruiz-Moreno E, Bermejo B, Antolín S, Montaño Á, et al. Clinical and Sociodemographic Determinants of Adherence to World Cancer Research Fund/American Institute for Cancer Research (WCRF/AICR) Recommendations in Breast Cancer Survivors-Health-EpiGEICAM Study. Cancers (Basel) [Internet]. 2022 Oct 1 [cited 2023 Jun 17];14(19). Available from: https://pubmed.ncbi.nlm.nih.gov/36230628/

10. Sandín B, Chorot P, Lostao L, Joiner TE, Santed MA, Valiente RM. Escalas PANAS de afecto positivo y negativo: Validacion factorial y convergencia transcultural. Psicothema. 1999;11(1).

11. Leyton M, Batista ;, Lobato ;, Jiménez Y, Ayudante P, Universidad D, et al. VALIDACIÓN DEL CUESTIONARIO DEL MODELO TRANSTEO?RICO DEL CAMBIO DE EJERCICIO FÍSICO. Revista Internacional de Medicina y Ciencias de la Actividad Física y del Deporte [Internet]. 2019 Jun 14 [cited 2023 Jun 17];19(74):329–50. Available from: https://revistas.uam.es/rimcafd/article/view/rimcafd2019.74.010

12. Díaz Fonte J, Cruzado JA, Díaz Fonte J, Cruzado JA. El Modelo Transteórico y el ejercicio en supervivientes de cáncer de mama. Clin Salud [Internet]. 2021 Nov 1 [cited 2023 Jul 7];32(3):129–37. Available from: https://scielo.isciii.es/scielo.php?script=sci_arttext&pid=S1130-52742021000300129&lng=es&nrm=iso&tlng=es

13. Requena GC, Salamero M, Gil F. Validación del cuestionario MOS-SSS de apoyo social en pacientes con cáncer. Med Clin (Barc). 2007 May 1;128(18):687–91.

14. Priede A, Andreu-Vaillo Y, Martínez López P, Ruíz Torres M, Hoyuela F, González Blanch C. Validación de la Escala MOS-SSS de apoyo social en una muestra de pacientes oncológicos recién diagnosticados. Proceedings of International Congress of Clinical Psycology. 2016;45–53.

15. Sociedad Española de Oncología Médica. Las cifras del cáncer en España [Internet]. 2025 [cited 2025 Feb 7]. Available from: https://seom.org/images/LAS_CIFRAS_DMC2025.pdf

16. Francis PA, Pagani O, Fleming GF, Walley BA, Colleoni M, Láng I, et al. Tailoring Adjuvant Endocrine Therapy for Premenopausal Breast Cancer. New England Journal of Medicine [Internet]. 2018 Jul 12 [cited 2023 Feb 18];379(2):122–37. Available from: https://www.nejm.org/doi/full/10.1056/NEJMoa1803164

17. Francis PA, Fleming GF, Láng I, Ciruelos EM, Bonnefoi HR, Bellet M, et al. Adjuvant Endocrine Therapy in Premenopausal Breast Cancer: 12-Year Results From SOFT. Journal of Clinical Oncology. 2023 Mar 1;41(7):1370–5.

18. Gray RG, Bradley R, Braybrooke J, Clarke M, Hills RK, Peto R, et al. Effects of ovarian ablation or suppression on breast cancer recurrence and survival: Patient-level meta-analysis of 14,993 pre-menopausal women in 25 randomized trials. Journal of Clinical Oncology [Internet]. 2023 Jun 1 [cited 2023 Jun 17];41(16_suppl):503–503. Available from: https://ascopubs.org/doi/10.1200/JCO.2023.41.16_suppl.503

19. Gradishar WJ, Moran MS, Abraham J, Abramson V, Aft R, Agnese D, et al. Breast Cancer, Version 3.2024, NCCN Clinical Practice Guidelines in Oncology. Journal of the National Comprehensive Cancer Network [Internet]. 2024 Jul 1 [cited 2025 Mar 6];22(5):331–57. Available from: https://jnccn.org/view/journals/jnccn/22/5/article-p331.xml

20. Loibl S, André F, Bachelot T, Barrios CH, Bergh J, Burstein HJ, et al. Early breast cancer: ESMO Clinical Practice Guideline for diagnosis, treatment and follow-up ☆. Annals of Oncology [Internet]. 2024 Feb 1 [cited 2025 Mar 6];35(2):159–82. Available from: https://www.annalsofoncology.org/action/showFullText?pii=S0923753423051049

21. Lu YS, Wong A, Kim HJ. Ovarian Function Suppression With Luteinizing Hormone-Releasing Hormone Agonists for the Treatment of Hormone Receptor-Positive Early Breast Cancer in Premenopausal Women. Front Oncol. 2021 Sep 14;11:3299.

22. Hojan K, Molińska-Glura M, Milecki P. Physical activity and body composition, body physique, and quality of life in premenopausal breast cancer patients during endocrine therapy--a feasibility study. Acta Oncol [Internet]. 2013 Feb [cited 2023 Feb 18];52(2):319–26. Available from: https://pubmed.ncbi.nlm.nih.gov/23193959/

23. Kauffman RP, Young C, Castracane VD. Perils of prolonged ovarian suppression and hypoestrogenism in the treatment of breast cancer: Is the risk of treatment worse than the risk of recurrence? Mol Cell Endocrinol. 2021 Apr 5;525:111181.

24. Friedenreich CM, Ryder-Burbidge C, McNeil J. Physical activity, obesity and sedentary behavior in cancer etiology: epidemiologic evidence and biologic mechanisms. Mol Oncol [Internet]. 2021 Mar 1 [cited 2022 Dec 11];15(3):790–800. Available from: https://pubmed.ncbi.nlm.nih.gov/32741068/

25. Demark-Wahnefried W, Schmitz KH, Alfano CM, Bail JR, Goodwin PJ, Thomson CA, et al. Weight management and physical activity throughout the cancer care continuum. CA Cancer J Clin [Internet]. 2018 Jan [cited 2023 Apr 25];68(1):64–89. Available from: https://pubmed.ncbi.nlm.nih.gov/29165798/

26. Giugliano F, Bertaut A, Blanc J, Martin AL, Gaudin C, Fournier M, et al. Characteristics, treatment patterns and survival of patients with high-risk early hormone receptor-positive breast cancer in French real-world settings: an exploratory study of the CANTO cohort. ESMO Open [Internet]. 2024 Dec 1 [cited 2025 Mar 6];9(12). Available from: https://www.esmoopen.com/action/showFullText?pii=S2059702924017642

27. Avgerinos KI, Spyrou N, Mantzoros CS, Dalamaga M. Obesity and cancer risk: Emerging biological mechanisms and perspectives. Metabolism [Internet]. 2019 Mar 1 [cited 2023 Mar 1];92:121–35. Available from: http://www.metabolismjournal.com/article/S0026049518302324/fulltext

28. Iwase T, Wang X, Shrimanker TV, Kolonin MG, Ueno NT. Body composition and breast cancer risk and treatment: mechanisms and impact. Breast Cancer Research and Treatment 2021 186:2 [Internet]. 2021 Jan 21 [cited 2022 Dec 11];186(2):273–83. Available from: https://link.springer.com/article/10.1007/s10549-020-06092-5

29. Zieff GH, Wagoner CW, Paterson C, Lassalle PP, Lee JT. Cardiovascular Consequences of Skeletal Muscle Impairments in Breast Cancer. Sports (Basel) [Internet]. 2020 [cited 2023 Feb 18];8(6). Available from: https://pubmed.ncbi.nlm.nih.gov/32486406/

30. Franceschi C, Garagnani P, Parini P, Giuliani C, Santoro A. Inflammaging: a new immune–metabolic viewpoint for age-related diseases. Nature Reviews Endocrinology 2018 14:10 [Internet]. 2018 Jul 25 [cited 2023 Feb 18];14(10):576–90. Available from: https://www.nature.com/articles/s41574-018-0059-4

31. Campbell KL, Winters-Stone KM, Wiskemann J, May AM, Schwartz AL, Courneya KS, et al. Exercise Guidelines for Cancer Survivors: Consensus Statement from International Multidisciplinary Roundtable. Med Sci Sports Exerc [Internet]. 2019 Nov 1 [cited 2023 Nov 20];51(11):2375–90. Available from: https://pubmed.ncbi.nlm.nih.gov/31626055/

32. Shaikh H, Bradhurst P, Ma LX, Tan SY, Egger SJ, Vardy JL. Body weight management in overweight and obese breast cancer survivors. Cochrane Database of Systematic Reviews [Internet]. 2020 Dec 11 [cited 2023 Feb 18];2020(12). Available from: https://www.cochranelibrary.com/cdsr/doi/10.1002/14651858.CD012110.pub2/full

33. Demark-Wahnefried W, Rogers LQ, Gibson JT, Harada S, Frugé AD, Oster RA, et al. Randomized trial of weight loss in primary breast cancer: Impact on body composition, circulating biomarkers and tumor characteristics. Int J Cancer [Internet]. 2020 May 15 [cited 2023 Apr 27];146(10):2784–96. Available from: https://pubmed.ncbi.nlm.nih.gov/31442303/

34. Schmidt T, Van Mackelenbergh M, Wesch D, Mundhenke C. Physical activity influences the immune system of breast cancer patients. J Cancer Res Ther [Internet]. 2017 Jul 1 [cited 2023 Apr 27];13(3):392–8. Available from: https://pubmed.ncbi.nlm.nih.gov/28862198/

35. Koelwyn GJ, Zhuang X, Tammela T, Schietinger A, Jones LW. Exercise and immunometabolic regulation in cancer. Nat Metab [Internet]. 2020 Sep 1 [cited 2023 Feb 18];2(9):849–57. Available from: https://pubmed.ncbi.nlm.nih.gov/32929232/

36. Ormel HL, van der Schoot GGF, Sluiter WJ, Jalving M, Gietema JA, Walenkamp AME. Predictors of adherence to exercise interventions during and after cancer treatment: A systematic review. Psychooncology [Internet]. 2018 Mar 1 [cited 2023 Apr 25];27(3):713. Available from: /pmc/articles/PMC5887924/

37. Ranes M, Wiestad TH, Thormodsen I, Arving C. Determinants of exercise adherence and maintenance for cancer survivors: Implementation of a community-based group exercise program. A qualitative feasibility study. PEC Innovation. 2022 Dec 1;1:100088.

38. Hojman P, Dethlefsen C, Brandt C, Hansen J, Pedersen L, Pedersen BK. Exercise-induced muscle-derived cytokines inhibit mammary cancer cell growth. Am J Physiol Endocrinol Metab [Internet]. 2011 Sep [cited 2023 Feb 28];301(3). Available from: https://pubmed.ncbi.nlm.nih.gov/21653222/

39. Michels N, van Aart C, Morisse J, Mullee A, Huybrechts I. Chronic inflammation towards cancer incidence: A systematic review and meta-analysis of epidemiological studies. Crit Rev Oncol Hematol [Internet]. 2021 Jan 1 [cited 2025 Feb 12];157. Available from: https://pubmed.ncbi.nlm.nih.gov/33264718/

40. Fiuza-Luces C, Valenzuela PL, Gálvez BG, Ramírez M, López-Soto A, Simpson RJ, et al. The effect of physical exercise on anticancer immunity. Nat Rev Immunol [Internet]. 2024 Apr 1 [cited 2025 Feb 12];24(4):282–93. Available from: https://pubmed.ncbi.nlm.nih.gov/37794239/
