# Supplementary material for: Randomized pilot study of an individualized multimodal exercise, nutrition, and behavior intervention in breast cancer patients treated with ovarian function suppression: protocol proposal for The OvS Breast ENBI Project
Source: Front Oncol. 2025 Oct 27;15:1622622. doi: 10.3389/fonc.2025.1622622 (PMC12597807; doi:10.3389/fonc.2025.1622622)
Supplement: Supplementary file 2 [file SupplementaryFile2.docx]

Supplementary Appendix 2

TIDieR Guide Recommendations. Exercise Intervention.

| **PROJECT NAME** | OvS Breast: ENBI Project |
| --- | --- |
| **OBJECTIVE** | A combined intervention based on physical exercise, nutritional management, and individualized psycho-oncological support may achieve weight control and preserve an adequate body composition in premenopausal women with localized BC expressing RE and/or RP undergoing adjuvant therapy with LHRH agonists in combination with TAM or AI. |
| **MATERIALS** | Free access will be provided to scheduled classes at a specialized exercise center for cancer patients, led by two certified exercise physiologists specialized in oncology.   - **Cardiovascular exercise:** Performed either in the park or at the cancer center, including step training, walking, and treadmill running, monitored with a heart rate monitor (1). - **Strength and neuromotor exercise:** Performed using medium-resistance elastic bands and free weights ranging from 2 kg to 20 kg (1) |

| **METHODS** | **Professionals** | Two certified exercise physiologists specialized in oncology with a degree in Physical Activity and Sport Sciences will carry out the assessments and intervention.   - One with 15 years of national and international experience in leading and developing oncology exercise programs and classes (2). - The other with 4 years of national and international experience leading group oncology exercise classes (2). |
| --- | --- | --- |
|  | **Location** | Specialized Exercise and Cancer Center. Outdoor exercise sessions will take place at El Retiro park (Madrid). |
|  | **Procedure** | A supervised program consisting of structured group sessions, individualized to each patient’s level, will be implemented. From the fifth session onward, these will be supplemented with home-based exercises (3, 13).  Patients will be assessed at baseline to determine their initial fitness level. Individual levels will be established using the interpretation tables described in ACSM’s Guidelines for Exercise Testing and Prescription ([link](https://shop.lww.com/ACSM-s-Guidelines-for-Exercise-Testing-and-Prescription/p/9781975219208?srsltid=AfmBOor30sydNl6jEDn2MVKjHZbBDIsWw8g727rd4RRF1w705H8HL59z)) (15) |
|  | **Type** | - **Cardiovascular exercise**: 15-50 minutes per session. - **Strength and neuromotor exercise**: at least 20 minutes per session. - **Stretching**: 10 minutes per session.   The training cycle (9, 1) will be repeated every 4 weeks, progressively increasing intensity to optimize adaptations and reduce injury risk.   - **Balance and joint mobility**: 30 min cardio including 10 min total of high intensity (distributed in different bouts) + elastic bands and proprioceptive circuit (8-12 exercises, 1-1.5 min each, combining upper limb exercises with elastic band exercises) + 10 min stretching. - **Global strength**: 20 min cardio including 6 min total of high intensity (distributed in different bouts) + global strength circuit (8-12 exercises, 2 x15 (40% RM) / 3 x10 (50-70% RM) / 8 x 40 s / 1 min each (40-50% RM maximal speed o over 50% RM) combining upper and lower limb strength exercises with various loads + 10 min stretching. - **Combined exercise with lower limb strength**: 10 min cardio including 8 min total of high intensity (distributed in different bouts) + combined strength and cardio circuit (8-12 exercises, 2 x15 (40% RM) / 3 x10 (50-70% RM) / 8 x 40 s / 1 min each (40-50% RM maximal speed o over 50% RM) ; 1-3 min of cardio) + 10 min stretching. - **Combined exercise with upper limb strength**: 10 min cardio including 8 min total of high intensity (distributed in different bouts) + combined strength circuit and 1 cardio exercise (8-12 exercises, same repetition schemes as above) + 10 min stretching.   Exercises Used (8)  **Cardio:** running, hill walking, walking; intensity assessed by HRR.  **Lower limbs**:   - Open squat ([link](https://www.youtube.com/watch?v=xqvCmoLULNY)) - Split squat ([link](https://www.youtube.com/watch?v=hXpGSa5HYqY)) - Lunge ([link](https://www.youtube.com/watch?v=wrwwXE_x-pQ)) - Cross lunge ([link](https://www.youtube.com/watch?v=wm_QY2Ym9kY)) - Deadlift ([link](https://www.youtube.com/watch?v=XxWcirHIwVo))   **Upper limbs and trunk**:   - Shoulder press ([link](https://www.youtube.com/shorts/aedn4SSc3Ww)) - Biceps curl ([link](https://www.youtube.com/shorts/SUEkPT8ufec)) - Dumbbell triceps extension ([link](https://www.youtube.com/shorts/3Bv1n7-DN7c)) - Dumbbell bent-over row ([link](https://www.youtube.com/shorts/IOOLhrkN_NI)) - Dumbbell grip row ([link](https://www.youtube.com/shorts/bH7iji2lTy0))   **Elastic-band exercises**:   - Hip adduction ([link](https://www.youtube.com/watch?v=Vce3tIYpzhc)) - Hip abduction ([link](https://www.youtube.com/watch?v=v-tN9LG547I)) - Banded glute bridge ([link](https://www.youtube.com/shorts/IoWia647VZY)) - Hip extension ([link](https://www.youtube.com/shorts/w0UNs9J89Lw)) - Trunk rotation ([link](https://www.youtube.com/shorts/876kdfHLd6c))   **Stretching**: [link](https://drive.google.com/file/d/1f_V7saLoMhKPc1IOLLr5pF6gYkuubX7d/view) |
|  | **Intensity** | Intensity will be progressively increased by adding 5% of the total time spent in high-intensity cardiovascular exercise every 3 weeks. Strength training load will be increased by 5% of total weight lifted per session every 2 weeks (7b).  If a patient fails to complete the prescribed intensity in the previous session, intensity will be maintained for one additional week. If the target is achieved, the following week the intensity will be increased (7a).   - **Cardiovascular exercise**: Performed at 55–100% HRR, monitored with a heart rate monitor and rated perceived exertion. - **Strength exercise**: Intensity assessed by total kilograms lifted per session (kg × reps) and % of RM, recorded relative to RM and rated perceived exertion using the Borg scale. - **Neuromotor exercise**: Execution time progressively increased from 40 seconds to 1.5 minutes. |
|  | **Frequency** | Two supervised sessions per week, plus one additional home-based session recorded via Polar Beat (4). |
|  | **Time** | 55-75 minutes per session (13). |
|  | **Intervention period** | 12 weeks (13). |
|  | **Pre-specified adaptations** | Exercise was tailored to each patient’s baseline assessment and perceived exertion (Borg scale). Additional adaptations included (14a, 14b):   - Rest period adjustments based on baseline level. - Exercise modifications for current or prior arthromyalgias. - Impact reduction for potential pelvic floor issues. - Intensity adjustments according to recovery capacity from previous session |
|  | **Adherence** | **Adherence will be assessed and reported through three variables (5, 16):**   - **Number of patients completing follow-up assessments.** - **Attendance to supervised sessions.** - **Relative dose intensity (percentage of planned sessions completed, percentage requiring dose reduction, and percentage of missed sessions)**  ([reference](https://pmc.ncbi.nlm.nih.gov/articles/PMC5953772/))   **To improve adherence, selected CALO-RE Taxonomy strategies (items 2, 5, 7, 12, 19, 27, 35, 38) will be integrated into exercise classes (6, 10).**  **Additional adherence-enhancing tools (6):**   - **Exercise supervised by oncology-specialized exercise physiologists.** - **Monitoring of home-based adherence via the free Polar Beat smartphone app (Android/iOS).** - **Tracking of attendance at face-to-face sessions and activities performed.** - **Monitoring of adverse effects and other events potentially interfering with sessions (e.g., sprains, muscle injuries, extreme outdoor temperatures).**   **Numbers in parentheses correspond to items from the Consensus on Exercise Reporting Template checklist (CERT). Items 11 and 16b were not included as they refer to events not yet recorded.** |

****Numbers in parentheses correspond to items from the Consensus on Exercise Reporting Template checklist (CERT). Items 11 and 16b were not included as they refer to events not yet recorded.***

TIDieR Guide Recommendations. Nutritional Intervention.

| **PROJECT NAME** | | OvS Breast: ENBI Project |
| --- | --- | --- |
| **OBJECTIVE** | | A combined intervention based on physical exercise, nutritional management, and individualized psycho-oncological support may achieve weight control and preserve an adequate body composition in premenopausal women with localized BC expressing RE and/or RP undergoing adjuvant therapy with LHRH agonists in combination with TAM or AI.  The nutritional intervention program aims to assess patients’ dietary habits at baseline and after the intervention, as well as to provide support throughout the process of change, with the objective of modifying their habits and improving their health. |
| **MATERIALS** | | **Individual 35-45 minutes sessions with the oncology nutritionistwill include the use of the following materials:**   - **Three-day dietary record.** - **Predimed questionnaire.** - **Compliance scale for WCRF/AICR recommendations.** - **Daily food intake questionnaire (form available via Google Forms).** |
| **METHODS** | **Professionals** | A specialist in oncology nutrition will carry out the nutritional intervention. |
|  | **Location** | Specialized Exercise and Cancer Center.en caso de necesidad. If required, the intervention will be conducted remotely (online). |
|  | **Procedure** | An evaluation of nutritional status will be performed before and after the intervention, based on the materials described. |
|  | **Frequency** | Three nutrition sessions will be conducted (weeks 4, 10, and 16) |
|  | **Time** | 35-45 minutes per session. |
|  | **Intervention period** | 12 weeks. |
|  | **Pre-specified adaptations** | - **Food allergy and intolerance accommodations** - **Making dietary recommendations suitable for vegan/vegetarian lifestyles** - Recommendations modifications based on diet-related conditions (e.g., cardiovascular risk factors such as hypertension, diabetes, or hypercholesterolemia) or digestive disorders/symptoms (e.g., inflammatory bowel disease, residual toxicities from oncological treatments such as bloating or heartburn). |
|  | **Adherence** | **The following tools are intended to enhance patients’ adherence to the program:**   - Working with a specialist in cancer nutrition. - Standardized and personalized nutritional intervention guidelines delivery. - Daily record supervision to determine whether changes established during the nutrition sessions have been followed. - Possibility of modifying the plan if any adverse effect occurs in relation to the indicated recommendations. |

TIDieR Guide Recommendations. Psycho-oncological intervention.

| **PROJECT NAME** | | OvS Breast: ENBI Project |
| --- | --- | --- |
| **OBJECTIVE** | | A combined intervention based on physical exercise, nutritional management, and individualized psycho-oncological support may achieve weight control and preserve an adequate body composition in premenopausal women with localized BC expressing RE and/or RP undergoing adjuvant therapy with LHRH agonists in combination with TAM or AI.  The psycho-oncological intervention program aims to increase motivation to achieve adherence to physical exercise program and nutritional recommendations, as well as to maintain long term healthy lifestyle habits. |
| **MATERIALS** | | **Three in-person sessions with a clinical psychology specialist and a resident psychologist (PIR). The following questionnaires will be used:**   - PANAS Affectivity Scale. - Transtheoretical Model of Exercise Change Questionnaire (CMTCEJ). - MOS Social Support Questionnaire. |
| **METHODS** | **Professionals** | This intervention will be conducted by two specialists in clinical psychology. |
|  | **Location** | Hospital General Universitario Gregorio Marañón Cancer Center. |
|  | **Procedure** | Three face-to-face sessions: an initial session for intervention and baseline assessment, an intermediate session for monitoring progress and achievement of objectives, and a final session to evaluate the changes achieved and their impact on mood and future outlook. |
|  | **Frequency** | Three psycho-oncological intervention sessions in weeks 4, 10, and 16. |
|  | **Time** | 90 minutes per session. |
|  | **Intervention period** | 12 weeks. |
|  | **Pre-specified adaptations** | Adaptation of the language and examples used in each session to the needs and prior repertoire of the patients. |
|  | **Adherence** | **The following tools are intended to enhance patients’ adherence to the program:**   - Encourage group participation by identifying the presence of shared goals. - Work on factors that contribute to group cohesion, such as creating safety, respect, and confidentiality place. - Promote patients’ self-observation and self-assessment. |
